# Open data and digital morphology

**DOI:** 10.1098/rspb.2017.0194

**Published:** 2017-04-12

**Authors:** Thomas G. Davies, Imran A. Rahman, Stephan Lautenschlager, John A. Cunningham, Robert J. Asher, Paul M. Barrett, Karl T. Bates, Stefan Bengtson, Roger B. J. Benson, Doug M. Boyer, José Braga, Jen A. Bright, Leon P. A. M. Claessens, Philip G. Cox, Xi-Ping Dong, Alistair R. Evans, Peter L. Falkingham, Matt Friedman, Russell J. Garwood, Anjali Goswami, John R. Hutchinson, Nathan S. Jeffery, Zerina Johanson, Renaud Lebrun, Carlos Martínez-Pérez, Jesús Marugán-Lobón, Paul M. O'Higgins, Brian Metscher, Maëva Orliac, Timothy B. Rowe, Martin Rücklin, Marcelo R. Sánchez-Villagra, Neil H. Shubin, Selena Y. Smith, J. Matthias Starck, Chris Stringer, Adam P. Summers, Mark D. Sutton, Stig A. Walsh, Vera Weisbecker, Lawrence M. Witmer, Stephen Wroe, Zongjun Yin, Emily J. Rayfield, Philip C. J. Donoghue

**Affiliations:** 1 School of Earth Sciences, University of Bristol, Life Sciences Building, Tyndall Avenue, Bristol BS8 1TQ, UK; 2 Oxford University Museum of Natural History, Parks Road, Oxford OX1 3PW, UK; 3 School of Geography, Earth and Environmental Sciences, University of Birmingham, Birmingham B15 2TT, UK; 4 Museum of Zoology, University of Cambridge, Downing Street, Cambridge CB2 3EJ, UK; 5 Dept. Earth Sciences, Natural History Museum, Cromwell Road, London SW7 5BD, UK; 6 Institute of Ageing and Chronic Disease, University of Liverpool, Liverpool L7 8TX, UK; 7 Dept. Palaeobiology, Swedish Museum of Natural History, PO Box 50007, 104 05 Stockholm, Sweden; 8 Dept. Earth Sciences, University of Oxford, South Parks Road, Oxford OX1 3AN, UK; 9 Dept. Evolutionary Anthropology, Duke University, PO Box 90383, Biological Sciences Building, 130 Science Drive, Durham, NC 27708, USA; 10 Computer-assisted Palaeoanthropology Team, UMR 5288 CNRS-Université de Toulouse (Paul Sabatier), Toulouse, France; 11 Evolutionary Studies Institute, University of Witwatersrand, Johannesburg, South Africa; 12 School of Geosciences, University of South Florida, Tampa, FL 33620, USA; 13 Center for Virtualization and Applied Spatial Technologies, University of South Florida, Tampa, FL 33620, USA; 14 Dept. Biology, College of the Holy Cross, Worcester, MA 01610, USA; 15 Dept. Archaeology and Hull York Medical School, University of York, York YO10 5DD, UK; 16 School of Earth and Space Science, Peking University, Beijing 100871, People's Republic of China; 17 School of Biological Sciences, Monash University, Victoria 3800, Australia; 18 School of Natural Sciences and Psychology, Liverpool John Moores University, Liverpool, UK; 19 Dept. Earth and Environmental Sciences and Museum of Paleontology, University of Michigan, Ann Arbor, MI 48109, USA; 20 School of Earth and Environmental Sciences, University of Manchester, Manchester M13 9PL, UK; 21 Dept. Genetics, Evolution and Environment, and Dept. Earth Sciences, University College London, Gower Street, London SW17 7PL, UK; 22 Structure and Motion Lab, Dept. Comparative Biomedical Sciences, The Royal Veterinary College, Hawkshead Lane, Hatfield, Hertfordshire AL9 7TA, UK; 23 Institut des Sciences de l'Evolution de Montpellier, CC64, Université de Montpellier, campus Triolet, Place Eugène Bataillon, 34095 Montpellier cedex 5, France; 24 Institut Cavanilles de Biodiversitat i Biologia Evolutiva, Universitat de Valencia, 46980 Paterna, Spain; 25 Unidad de Paleontología, Dpto. Biología, Universidad Autónoma de Madrid, 28049 Cantoblanco, Spain; 26 Dept. Theoretical Biology, University of Vienna, Althanstrasse 14, 1090, Austria; 27 Jackson School of Geosciences C1100, The University of Texas at Austin, Austin, TX 78712, USA; 28 Naturalis Biodiversity Center, Postbus 9517, 2300 RA Leiden, The Netherlands; 29 Paläontologisches Institut und Museum der Universität Zürich, Karl Schmid Strasse 4, 8006 Zürich, Switzerland; 30 Dept. Organismal Biology & Anatomy, University of Chicago, Chicago, IL 60637, USA; 31 Dept. Biology II, Ludwig-Maximilians University Munich (LMU), Großhadernerstr. 2, 82152 Planegg-Martinsried, Germany; 32 University of Washington, Friday Harbor Labs, Friday Harbor, WA 98250, USA; 33 Dept. Earth Science and Engineering, Imperial College, London SW7 2AZ, UK; 34 National Museums Scotland, Chambers Street, Edinburgh EH1 1JF, UK; 35 School of Biological Sciences, The University of Queensland, St Lucia, Queensland 4072, Australia; 36 Dept. Biomedical Sciences, Ohio University Heritage College of Osteopathic Medicine, Athens, OH 45701, USA; 37 School of Environmental and Rural Science, University of New England, Armidale, New South Wales 2351, Australia; 38 State Key Laboratory of Palaeobiology and Stratigraphy, Nanjing Institute of Geology and Palaeontology, Chinese Academy of Sciences, Nanjing 210008, People's Republic of China

**Keywords:** digital data, three-dimensional models, phenotype, computed tomography, visualization, functional analysis

## Abstract

Over the past two decades, the development of methods for visualizing and analysing specimens digitally, in three and even four dimensions, has transformed the study of living and fossil organisms. However, the initial promise that the widespread application of such methods would facilitate access to the underlying digital data has not been fully achieved. The underlying datasets for many published studies are not readily or freely available, introducing a barrier to verification and reproducibility, and the reuse of data. There is no current agreement or policy on the amount and type of data that should be made available alongside studies that use, and in some cases are wholly reliant on, digital morphology. Here, we propose a set of recommendations for minimum standards and additional best practice for three-dimensional digital data publication, and review the issues around data storage, management and accessibility.

## 1. Introduction

Three-dimensional (3D) digital morphological data are commonly employed by palaeontologists and biologists in research. In palaeontology and anthropology, the widespread application of tomography (especially X-ray computed tomography, CT), laser and structured light scanning, and photogrammetry has revolutionized the study of morphology [1–4]. In biology, optical microscopy, magnetic resonance imaging (MRI) and contrast-enhanced CT are important tools for investigating soft-tissue anatomy [5–10]. The revolution brought about by these technologies has increased the amount and detail of anatomical information recovered from fossil and living organisms, transforming the nature of scientific enquiry in related fields. The resulting datasets are often reconstructed and presented as 3D digital models, which are themselves sometimes used in downstream analyses, including geometric morphometrics [11,12], finite element analysis (FEA) [13], multibody dynamics analysis (MDA) [14] and computational fluid dynamics (CFD) [15], thereby facilitating quantitative tests of functional and evolutionary hypotheses [3]. These types of studies have yielded important advances in our understanding of the anatomy of living and fossil organisms (e.g. [10,16,17]), as well as fundamental aspects of their biology, from feeding mode [18–20] to mobility [21,22], development [23,24] and physiology [25–27], as well as developments in taxonomic practice [28,29]. Barriers to data sharing and access to specimens can be eroded because data exist as digital files that can be easily copied and readily distributed, allowing simultaneous analysis by multiple researchers [30]. These attributes should also enhance the verifiability and reproducibility of studies, facilitating the reuse of data and metadata, more in-depth interrogation of any given dataset, and broader-scale comparative analyses through the assembly of large datasets of multiple specimens or taxa.

However, authors of studies involving 3D digital datasets of biological and palaeontological specimens often do not publish their supporting data, meaning that results and conclusions cannot easily be verified or replicated, and that this potentially valuable source of novel data cannot be further explored [30]. Ultimately, digital data collected but unpublished are likely to be lost to science [2,28]. This also represents a substantial waste of financial and other resources, and places vulnerable original specimens at greater risk of damage or loss, as the same specimens are likely to be reimaged repeatedly to enable different groups of workers to reproduce the data [28,31]. Consequently, the promise of 3D digital data has not yet been fully realized.

This is not news [2,28,30]. However, most national and international funders have imposed regulations on data access and sharing that are forcing researchers and institutions to finally confront this challenge [32]. These regulations range from funder-mandated full release of all data [32], through declarations that the data are available from authors on request, to no release of supporting data [32]. When data are released, they are deposited in a diversity of online databases (e.g. BIRN, Dataverse, Dryad, EOL, figshare, GigaDB, Github, MorphoBank, MorphoDBase, MorphoMuseuM, MorphoSource, Phenome10 K, Zenodo), institutional and funder repositories, physical museums, and research group websites. At least in part, this diversity of approaches reflects uncertainty about the available repositories for data deposition and the cost of storing the comparatively large files associated with digital imaging-based research. Researchers can also be reluctant to share data that remain part of an active research programme [33], or to share a subset of data that is part of a larger, unpublished package. There is also a lack of consensus and widespread confusion over issues of data ownership and copyright, and conflict that emerges between institutional policies asserting copyright ownership (e.g. public museum or even private collections) and the regulations of funding bodies and publishers with regard to open data. Consequently, sharing or publishing supporting data is often a low priority and has effectively been considered optional when not prescribed by a journal. Partial datasets (e.g. low-resolution visualizations or external surfaces) can be insufficient for reproducibility or even verification. As digital morphology has evolved, most of us in the research community have failed to achieve what might now be considered best practice of open data.

The academic world has already taken important steps towards overcoming some of these motivational and practical obstacles. Platforms for both archiving and sharing data online are becoming more commonplace, and can handle large file sizes. The standard in molecular biology is GenBank (https://www.ncbi.nlm.nih.gov/genbank/), where sequence data underpinning studies are accessioned before publication. For other data formats, journals and publishers offer a mixed landscape of policies on data publishing that is in need of standardization [34,35], but many not only mandate data deposition—some are even prepared to bear the associated costs, making data deposition easier and ultimately improving science, both in terms of practice and accessibility. There are also initiatives to integrate data submission with submissions to peer-reviewed journals, requiring (or at least allowing) the submission of data in the article submission process and enabling reviewers to examine supporting data as part of the review process [36]. However, collectively, these initiatives have not been integrated [34], and they have not yet translated into common practice within many subdisciplines in biology, palaeontology and anthropology.

If a consensus can be established among authors, repositories, journal editors, peer reviewers and funding agencies, there is the prospect of finally realizing the potential of digital morphology in the open-data era. Here, we make recommendations on the nature and extent of essential and recommended best practice datasets that should be made available to support scientific publications using 3D digital datasets across biological sciences (summarized in tables 1 and 2). We review the requirements of associated metadata, discuss the current range of repositories available for such studies and comment on issues affecting their utility.

**Table 1.** Summary table of recommendations for types of data files that should be published in support of published articles. Everything in the ‘essential’ column must be provided to enable reproduction of the study (assuming the information about how the 3D model was produced is sufficiently detailed). By contrast, the ‘recommended’ column represents our suggestions for improving the transparency of the process and should be provided where possible (i.e. when storage space is not a major problem, like in studies based on scans of single specimens). 3D models should be provided at the resolution at which analyses are conducted.

mode	imaging method	essential (for verification)	recommended (as best practice)
3D models	tomography	—full-resolution image stack (e.g. TIFF) —final 3D models used in study (e.g. STL) —text file with description of scan settings^a^, voxel size, techniques used to produce 3D models, and specimen information (e.g. copyright, repository, and accession number)	—prepared dataset (i.e. segmented images) consisting of image stack and/or project folder (e.g. Avizo label fields, SPIERS masks) —unregistered image stack (for physical and optical tomography)
	laser or structured light scanning	—final 3D models used in study (e.g. STL) —text file with description of scanner settings, resolution, techniques used to produce 3D models, and specimen information (e.g. copyright, repository, and accession number)	—3D models retaining texture information^b^ (e.g. PLY or OBJ) —original capture data (i.e. data acquired by scanner)
	photogrammetry	—final 3D models used in study (e.g. STL) —text file with description of how images were acquired, scale, techniques used to produce 3D models, and specimen information (e.g. copyright, repository, and accession number)	—3D models retaining texture information^b^ (e.g. PLY or OBJ) —original capture data (i.e. photographs)
additionally for downstream functional analyses:			
morphometrics		—landmark coordinates and rules defining automated landmark capture —images used in 2D landmark analysis (e.g. TIFF) —3D models used in 3D landmark analysis (e.g. STL) —text file with description of how analysis was performed and specimen information (e.g. copyright, repository, and accession number)	
functional analyses		—3D models used in functional analysis —project file with details of material properties and boundary conditions used in analysis	—project file with results

^a^This should include: details of the scanner, current, voltage, number of projections, exposure time and filter thickness (if any).

^b^Essential if critical to the analysis.

**Table 2.** Summary of the principles of open data for digital morphology.

data publication
—all the data required to replicate and verify a published study must be made available immediately upon publication —published data must include original image stacks (for tomography), final 3D models (for tomography and surface-based methods), landmark data (for morphometrics), and files containing details of the analysis set-up and parameters (for functional analysis); metadata outlining how these data were collected and processed, together with information on copyright and details of the original specimens under study, must also be provided —additionally, as best practice, original capture data (for surface-based methods), unregistered images (for optical and physical tomography), prepared datasets (for tomography) and results files (for functional analysis) should be provided —data files should ideally be published in widely accessible standard formats, such as TIFF for image stacks, STL or PLY for 3D models, and TXT for metadata; however, where no standard format exists (e.g. many functional analyses), proprietary file formats may be used

data storage
—data underlying a published study must be deposited in a suitable repository —data repositories should guarantee the preservation of data in their published form indefinitely, while also facilitating easy access; moreover, repositories should ensure that a unique and persistent identification code (e.g. DOI) and all relevant metadata are associated with the published data —data should be published under a standard copyright licence (e.g. creative commons), and the licence chosen (e.g. CC-BY, CC-BY-NC) should enable the greatest use by the widest possible audience, while still respecting genuine concerns over ethical issues and commercial activities; depending on the licence under which the data were published, a system for monitoring data access and/or usage (e.g. digital watermarking) could be implemented —data producers should devise a strategy for meeting the costs of long-term data storage (e.g. applications for external funding) at an early stage in their research; in some cases, costs may be minimized by reducing file sizes using lossless data compression

data reuse
—data producers should provide a statement of intent outlining how they intend to exploit their published dataset over a short specified time frame (e.g. six months to 1 year); other researchers are free to reuse these data for other purposes immediately following publication and for any purpose (within the restrictions of the copyright licence) after the conclusion of this stated time frame —data users should contact data producers to discuss research plans in case of overlapping interests; where appropriate, this may include collaborative projects leading to joint outputs (e.g. publications) —data users must credit the original published dataset upon reuse; journal editors and reviewers should ensure that this practice is correctly followed in all relevant publications

## 2. Publishing tomographic data

A range of methods exist for studying 3D specimens through the creation of two-dimensional (2D) image stacks (i.e. tomography), including X-ray CT (encompassing medical CT, micro-CT and synchrotron tomography), MRI, neutron tomography, optical tomography, histological microtomy and physical tomography [1,3,4,37,38]. All of these techniques generate datasets consisting of up to several thousand parallel sections or slices (tomograms) through a specimen, with each tomogram represented by an image file. Various techniques exist for the construction of 3D digital models from sets of tomograms [1].

### (a) Data essential for scientific verification

#### (i) The image stack

Image stacks are the starting point for most tomographic studies. These provide immediate insight into internal and external features, and form the basis for any subsequent construction of 3D models. Image stacks exist in a range of non-proprietary file formats, but the most common include DICOM, TIFF, JPEG, PNG, VOL, RAW and BMP [39]. All such files can be opened and viewed in free software such as ImageJ, Drishti, SPIERS, Horos and 3D slicer [40], and can be converted into different formats, although this can be more difficult with DICOM files, which exist in a multitude of sub-formats, not all of which can be handled by all software. For most purposes, TIFFs (16- or 8-bit) provide the best balance of accessibility, file size and data quality (lossless compression), but any lossless, standard image file-types are sufficient. Most JPEG formats enforce a lossy compression scheme that may degrade over multiple save operations; lossless JPEG formats do exist (JPEG-LS, JPEG 2000), but they are not widely used. These differences underlie the importance of specifying the file standard used [39]. Minimally, image stacks should retain the contrast resolution (bit-depth) and spatial resolution used in the study. In cases where the image stack is derived from K-space filling (e.g. MRI) or a series of angular projections (e.g. X-ray CT), the process of generating the image stack is largely automated and we do not consider it necessary to publish the raw projections.

#### (ii) Metadata

An image stack alone will not contain all the information necessary to make full use of the data. For example, scale is only preserved if the resolution (e.g. voxel size or slice spacing) is encoded in the files, and for some datasets slice spacing is not constant and requires per-slice documentation. In the case of DICOMs, this information is typically retained within the file or can be added to the file with a header tag editor (e.g. ImageJ). Otherwise, a text file detailing the voxel or pixel size and slice spacing is the minimum necessary information that must accompany publication of any image stacks. Additionally, metadata information should include full details of how the images were acquired (including scan settings), and further information on data copyright, repository and accession of specimens scanned and, if appropriate, comments on preparation or specimen storage for biological specimens (table 1). This information is necessary to reproduce studies, as well as to evaluate if better-quality data could be obtained with a different set of parameters [41]. Minimally, these data should be provided in a simple text file (e.g. TXT or VGI) associated with the dataset, regardless of whether the information is provided in any study based on the data.

#### (iii) Three-dimensional models

Typically, tomographic studies involve the reconstruction of 3D models from image stacks, in some cases after image segmentation or other preparation (see below). 3D models are normally triangle-mesh geometries generated via isosurfacing (usually known as surface models) [1]. Publication of the 3D models resulting from isosurfacing allows for the interactive examination of specimen morphology in three dimensions. A wide range of free software is available for this task [1,3], although no ideal general-purpose file format exists for complex models (see below). 3D models may have been modified after initial isosurface construction, for example through smoothing, island removal or hole filling. Consequently, the most appropriate model to publish to enable verification is the final model (or models) on which the results of the study are based, or which is used in downstream analyses.

The 3D models generated using tomographic data are available in a range of different file formats [1,42]. The choice of file type may be influenced by various factors including file size and whether colour/texture information is required; it is essential that openly accessible, standard formats are used (e.g. STL, PLY or OBJ), but there is no single ‘ideal’ file format. The stereolithography (STL) format is the most widely used standard for publishing 3D triangle meshes derived from tomographic techniques, and it is simple and supported by the vast majority of 3D visualization programs, including freely available software [1]. STL files are also compatible with most modern 3D printers, offering potential for wider applications in specimen conservation, public outreach or teaching [3,43]. However, STL files cannot store data on colour, texture or scale. Where these are an essential part of the study, an alternative format such as PLY, OBJ with MTL or VAXML [1,39,42] will be required. These formats are also recommended for meshes with a high number of triangles, which can result in very large file sizes in the STL format.

### (b) Additional data required for best practice

#### (i) Prepared datasets

While some tomographic datasets are reconstructed as 3D models without any modification or markup, this is unusual. Most datasets are subjected at least to segmentation, the semi-automated or manual differentiation of voxels (3D pixels) into distinct regions-of-interest (using, for example, ‘label fields’ in Avizo or ‘masks’ in SPIERS). Some datasets also require semi-automated or manual modification of the data (e.g. through brightness modifications) to better separate specimen from background (we term this ‘editing’). These processes involve a degree of subjective interpretation; this is especially true for palaeontological datasets, which are often very noisy and can require extensive manual intervention to extract maximal information from the original data. Thus, publication of the original tomographic dataset and final 3D model may not be sufficient to enable other researchers to assess the association between the two. Segmenting and/or editing a tomographic dataset can be very time-consuming and therefore difficult to reproduce in practice; without access to prepared datasets, most secondary users would not be able to fully interrogate the data underlying a 3D model. In such instances, prepared datasets should be released. No standard file format exists, but labels and masks can be released in the native formats by the software used to generate them, or as binary image stacks, which can then be readily reconstructed as a 3D model in a variety of software packages [1,42].

Development of back-projection algorithms can improve signal to noise ratio in generated image stacks and, hence, recent open-data mandates at synchrotron facilities require archiving of the radiograph projections, not the resulting slice data [44]. Thus, it may be sensible for authors to archive the raw projection libraries themselves. This is especially important where access to the same specimen may be problematic, or as a precaution in case unique specimens are damaged, lost or destroyed.

#### (ii) Image registration

For physically destructive and optical tomography, tomograms need to be registered (aligned relatively and absolutely in the X, Y and Z planes, either manually or semi-automatically) prior to any reconstruction of 3D models. This adds a potentially subjective step that may have a bearing on downstream analyses, and so we recommend publishing both the original (unregistered) and registered image stacks as best practice.

## 3. Publishing three-dimensional data from surface-based methods

Alternative surface-based methods exist for digitizing only the exterior features of specimens in 3D, most notably laser or structured light scanning [45] and photogrammetry [1,46,47]. For photogrammetry, data begin as 2D photographs, whereas in surface-scanning techniques, the 3D shape is usually directly captured as 3D point clouds, with or without texture capture (colour) for each point. In photogrammetry, a 3D polygonal mesh with texture data is generated and warped onto the 3D surface (typically automatically), giving each triangle a colour value. Scanning methodologies may directly visualize point clouds, or may generate and visualize a 3D triangle mesh, with or without texture mapped onto triangles or vertices.

### (a) Data essential for verification

#### (i) Three-dimensional models

The production of the initial 3D surface from photographs or surface scans is largely automated. The most critical data are the final 3D surface files, which may be fused from the original component meshes (e.g. in STL, PLY or OBJ formats) [39]. In cases where the surface texture (i.e. colour information) is directly relevant to the outcomes of a study, the published 3D models must retain this information (i.e. should be provided in PLY or OBJ formats). Surface models are not normally segmented into multiple geometric objects, so single-file models in PLY or STL format are practical.

#### (ii) Metadata

A text file of metadata should be provided that documents details of the imaging settings and techniques used to generate the 3D model (table 1). Preparation of 3D meshes may involve a range of operations, including trimming irrelevant data, realigning or reorienting components of the mesh, fusion into a single mesh, smoothing, hole filling and/or manual manipulation of the location of individual point coordinates or surfaces. These operations should be detailed in the metadata file. Where such operations are non-trivial and/or involve interpretation, those data (photographs, raw point clouds) are an essential provision, in open and widely accessible formats, where possible.

### (b) Additional data required for best practice

#### (i) Models including texture information

Colour data from the surface can provide useful information to help interpret the specimen (e.g. taphonomic preservation). As best practice, this should be included if available, in PLY or OBJ format.

#### (ii) Original capture data

The photographs or data captured by the scanner or the 3D data generated by the photogrammetry software allow verification of the processes used to generate the model and should be included as best practice. For 3D scanning, in some cases it may only be feasible to release the raw data in proprietary formats but, where possible, widely compatible (e.g. STL) surfaces should be exported. For methods that involve the digital alignment of different aspects of a specimen, or significant manual intervention in the model construction, the unfused data should be released as the accuracy of the original alignment may be of variable quality.

## 4. Downstream analyses (morphometric and functional analyses)

It is important to consider not only the generation of 3D models, but also the data that may be produced in the course of downstream analyses to which these data are subjected. Common types of analysis include: (i) size and shape analyses through topological and landmark-based techniques such as geometric morphometrics; and (ii) assessment of the functional performance of specimens through computer modelling approaches, such as FEA, multibody dynamics analysis (MDA) or CFD. These studies are often based on 3D models with the data subsequently analysed in specialist software packages [1].

### (a) Data essential for verification

#### (i) Morphometric data

For morphometric approaches, the original landmark coordinates and the rules defining landmark location should be provided as these constitute the raw data for the morphometric analyses. For 2D landmark data, a TPS file or similar format links landmarks to their constituent images. Where 3D landmark data points are collected via a 3D digitizer, it is common practice to tabulate the specimen number of the digitized specimen. Where the analyses are based on 3D surfaces or digital models, it is desirable that the models (surface or volume) used in the analysis should be published in an accessible format (following the guidelines outlined above).

#### (ii) Downstream functional data

Functional analyses typically convert 3D digital datasets into proprietary formats for specific methodologies, such as FEA, CFD and MDA. Free software packages do exist, but typically industry standard commercial packages are employed. These have the advantage of reliability and standardized algorithms underpinning the computational analysis.

#### (iii) Project files or metadata

Specialist software has the disadvantage that it outputs data in proprietary file formats that may not be widely accessible to many potential users. For morphometrics, a text file detailing any corrections or transformations applied to the data and an explanation of the analyses should be published. If the morphometric analysis is conducted in the R environment, an annotated R script is a convenient solution. For 3D functional analyses, the (usually proprietary) files containing the analysis set-up and parameters, either with or without the results files, are required for model verification. This addition enables a user with access to the appropriate software to replicate the analyses. Full metadata should be provided with details of processing techniques used to generate the final model, as well as a description of any parameters specified by the user in the analysis (table 1).

### (b) Data required for best practice

#### (i) Project and results files

Analytical techniques used to investigate the function and biomechanical performance of 3D modelled taxa will produce a range of additional digital data, which should also be made available in order to replicate studies. In the case of FEA, programs use volumetric meshes consisting of a finite number of elements. For MDA and CFD, formats such as the parasolid standard are often essential to perform the analyses. Further parameters and boundary conditions are then defined in specialist software (e.g. Abaqus, Ansys, Strand 7, Adams, Opensim, Gaitsym, COMSOL). Ideally, both the model set-up as well as the result files would be published alongside a study. For commercial packages, viewing software is sometimes available which allows the display of models and results files, but no additional analyses. Some industry software packages have text-editor-readable files that list and detail the location and nature of boundary conditions (e.g. INP files for Abaqus FE software).

## 5. Data repositories

Researchers have a responsibility to ensure that all of the data necessary to reproduce a published study are made available. As explained above, for 3D digital datasets these data may include original 2D images, prepared/segmented 3D images, 3D geometries and relevant metadata. These datasets can be, *in toto*, very large by today's standards; over 100 GB per specimen is possible in some scenarios, and there may be some instances where single publications utilize huge numbers of specimens, the storage of which is in itself a project. Publishers and other institutions hosting repositories must manage and facilitate access to the data they host, with these obligations persisting into the future, ideally indefinitely. Museums and other institutions holding original specimens often consider digital data as an intrinsic aspect of the specimen, and request researchers to deposit these data with them. Many have active programs of 2D and 3D digital curation, and normally make data freely available for research purposes. Data access for commercial use is a source of much-needed income, and commercial reuse of data released for research purposes is a genuine concern. However, most museums do not yet have systems, policies or resources in place for the long-term curation and distribution of digital morphological data [30]. This is not surprising given the paradigm shift in the concept of the accessioned specimen brought about by digital morphology, expanding from the physical specimen to a diversity of avatars.

Digimorph.org pioneered the curation of digital morphological data for in-house scans generated by the University of Texas High-Resolution CT Facility (UTCT), and there are now a number of general and specialist repositories facilitating the publication and dissemination of supporting data at a variety of scales (electronic supplementary material, table S1). Many journals have agreements with such repositories and will cover charges, even for relatively large datasets. In addition, many funding agencies cover the costs of long-term data storage, and many institutions have developed their own data repositories to manage research data generated by their own researchers. Out-moded promises to make data ‘available on request’ should give way to permanent URL links to 3D image data in biology, anthropology and palaeontology (cf. [35]).

### (a) Available data repositories

A range of repositories are available that cater for 3D digital datasets arising from research in biological sciences (electronic supplementary material, table S1). These can vary greatly in terms of the size and types of data they are willing to accept, as well as the cost of storage. In some cases, the choice of repository may be prescribed by the funding body or journal, but this decision will most often be made by the researcher. Modern facilities for publicly sharing datasets include national data centres (typically supported by a research funding body; e.g. RCUK data centres), multidisciplinary (e.g. Dryad, datadryad.org; figshare, figshare.com; MorphoMuseuM, morphomuseum.com; MorphoSource, morphosource.org; Phenome10 K, phenome10 k.org; Zenodo, zenodo.org) or discipline-specific (e.g. XROMM, xromm.org) repositories, and institutional repositories for data produced in-house (e.g. Bristol University's Research Data Repository, data.bris.ac.uk/data; Natural History Museum London's Data Portal, http://data.nhm.ac.uk). It is not entirely clear that all of these are sustainable in the long term. Traditional repositories of physical specimens can also store and disseminate data, and many are moving towards online access to their digital collections.

### (b) Necessary standards for data repositories

Digital repositories should have the same qualities as repositories of physical specimens, in that they should ensure the long-term persistence and preservation of datasets in their published form, provide expert curation and stable identifiers for submitted datasets, and facilitate public access to data without unnecessary restrictions. However, by their very nature, they should also ensure that the data are discoverable online, provided with unique, permanent and citable reference codes (e.g. DOIs), associated with relevant metadata (e.g. readme text file), and have links to relevant publications and funding bodies [2,28].

The specific licence used by the repository should be considered. Many facilities currently use the CC-BY-NC licence, which disallows reuse for commercial activities. This may be desirable where there are concerns over activities such as selling 3D prints of museum specimens with no benefit to the institutions charged with maintaining those collections. Some data repositories (e.g. MorphoSource) allow users to specify the most appropriate licence for their data. Authors may prefer to choose the CC-BY licence, which is among the most open creative common licences available and has become the standard for open access publication of journal articles. This licence lets others distribute, edit and build upon the original data, even commercially, as long as they credit the original creator. The CC-0 licence (Dryad default) goes further and allows copyright owners to waive all rights. CC-BY-ND is less attractive, as it allows sharing but does not allow the end user to publish derivatives of the data.

3D digital datasets associated with published studies should be verifiable and fully traceable from production to publication, and later republication. One option is digital watermarking, which provides a means of achieving verification of the authenticity and integrity of data, and is imperceptible to the human eye, but also durable in both digital and printed forms, surviving most image edits, file format conversions, data compression, filtering, partial data removal and smoothing. Another option would be to require users to register with the repository before data can be downloaded and used, a practice already imposed by some repositories (e.g. Dryad, MorphoSource). Registration is usually free and open to everyone, but allows the repository to track data access.

### (c) Costs

When publishing large (e.g. more than 10 GB) 3D digital datasets, it is vital to consider the financial costs, which are typically proportional to the amount of data being stored. Some repositories do not currently charge for accessions (e.g. MorphoSource), but for some, accession charges are not insignificant. The popular online digital repository Dryad (datadryad.org) currently charges $120 per data package of 20 GB plus $50 for each additional 10 GB. Datasets based on synchrotron tomography supporting a single publication can easily run to 100 GB for a relatively small number of scans of individual specimens, and it is possible to envisage future projects, especially synthetic papers and large-scale comparative analyses, generating datasets that are orders of magnitude greater in size. Publishing such datasets can quickly become prohibitively expensive; many journals offer to fully or partially cover the costs of depositing digital datasets, but do not have a clear policy for datasets that are hundreds of GB to TB in size. Applications for research funding are increasingly budgeting for data storage costs, but this does not assist projects making use of pre-existing data, or those where funds for data publication are not available.

One way of minimizing costs is by reducing the total size of data published without compromising the quality. Cropping of redundant space around a volume representing the specimen is an obvious first step. Lossless compression of individual image files is an excellent route to reduce data storage for image stacks in certain formats. For example, LZW compression, both lossless and fully reversible, can provide upwards of 40% reduction in file size on eight-bit TIFFs with no evident effect on data quality, but it is not routinely applied. The PNG image format provides a similar level of lossless compression. Most of the JPEG image formats enforce lossy compression that degrades data, and should not be used despite appealingly high compression ratios. Placing files into ZIP archives (e.g. one ZIP file per image stack) also reduces disc space through lossless compression and is more convenient for downloading. However, ZIP and VOL archives are less secure for long-term storage since, if the single file containing a dataset becomes corrupted, the entire dataset will be lost. Corruption of single files within a large dataset is less serious, and at least some repositories have procedures in place to detect and remediate bitrot [31]. We recommend that unarchived copies of the original data are stored and made available where possible.

In our enthusiasm for recycling 3D digital data and easing reproducibility of morphological studies based on them, the environmental costs of storage should be considered. Most datasets will be accessed infrequently and so there is no need or justification for their storage on spinning discs. Many repositories make use of automated tape storage which is stable and comparatively low in direct costs for the same reasons that make it environmentally low-cost.

## 6. Rescuing legacy data and constraints on data use

An increase in the availability and ease of use of data repositories raises the prospect of making data available from previously published studies where the data were not released at the time of publication. Digital datasets can be uploaded to online data repositories and linked to past publications. At present, there are no policies or mechanisms we are aware of among journals and publishing houses to link archival publications to newly deposited data. However, there is no material technical barrier to salvaging legacy data in this way. Publishers are likely to welcome such an initiative as it would obviously improve data visibility, facilitate reproducibility, and probably rejuvenate old publications in terms of access, citations and, ultimately, their marketability.

Obtaining digital characterizations of morphology can be time-consuming and expensive, and researchers rarely exhaust their data with the first publication. Funders and publishers are increasingly removing choice over whether to release supporting data, and so it can seem unfair that the researchers who generated datasets have to subsequently compete to exploit them further. This can be particularly difficult for lone early-career researchers potentially competing with large experienced research groups [33]. One potential solution to this would be the introduction of time-limited embargos, which can already be facilitated by some data repositories. However, such embargos violate the most basic tenet of open data: that of removing barriers to assessing the reproducibility of research [48]. After the point of publication, it is also effectively impossible to police the release of supporting data and, consequently, we see no alternative to the release of data with publication. A possible compromise may be borrowed from the Bermuda [49], Fort Lauderdale [50] and Toronto [51] agreements of the genomics community. These mandate data release at the time they are obtained but, more germane to morphologists, these agreements provide safeguarding for data generators through published, time-limited statements of intent of how they propose to exploit the data [51]. Other researchers are free to exploit the data for other purposes, and for any purpose after the stated period of limitation of the statement of intent [52]. Third-party users with overlapping research interests are expected to proceed respectfully and in dialogue with the data generators to identify a mutually agreeable publication schedule [51]. Invariably, much more is at stake in such projects, and though these informal agreements are rarely violated, they are generally well policed by the peer review process [52], and by the reputational damage suffered by those who choose not to observe these agreements.

Practice in the genomics community underscores the point that there is more to gain from open data than the warm glow of altruism [51,53]. Not only has it led to greater and more rapid scientific advance [48,51], it can lead to material personal gain, through proposals for collaborative exploitation of published data, both to achieve stated research objectives, and to achieve new objectives that would not be possible without unforeseen collaborators [51,53]. Citation and access-tracking of published datasets also provide credit to the authors [31]. Attribution of authorship is mandated under CC-BY licences and is in any case integral to the academic culture. Many journals already mandate citation of published datasets, not (or not merely) the publications describing research based upon them; this must become common practice. Further mechanisms for encouraging researchers to share their data should only add to this motivation, such as explicitly evaluating the open sharing of data in hiring, promotion or other reward processes.

Nevertheless, data can be associated with ethical sensitivities that may require the withholding, or restriction on public distribution, of data (e.g. anthropology or medical science [54,55]). In such instances, the issues that apply should be clearly defined so that beyond these boundaries researchers and publishers can follow an ethos of open-data publication. Mechanisms already exist to cope with these constraints while still making data available, such as data anonymization and vetted access [51].

## 7. Outstanding challenges

While the principle of open data has been mandated by the majority of funders [32], publishers, physical repositories and researchers are all scrambling to meet the resulting challenges. Above all, the competing interests over ownership of digital data need to be resolved between (i) funders who pay for research, (ii) researchers who collect specimens and create the digital datasets, (iii) research facilities where data are collected, (iv) museums that have a duty of care for the physical specimens and (v) research publishers. Funders, researchers and publishers may have converged on an ethos of open data. However, the institutions that are responsible for the physical specimens have not obviously been invited to engage in the development of open-data policy, and yet it is museums that will have to change most in terms of their policies on the nature of what they consider intrinsic aspects of the physical specimens that they hold in their care. One solution for museums might be to comply with research funders’ requirements, and waive copyright over digital representations of their collections, along with its associated income stream. Another solution would be for these institutions, which are those best-placed to inform policy on the curation, storage and distribution of data, to develop digital collections with the stability to match that of their physical inventory. Indeed, with the development of cybertypes [28,29], this may be an inevitable future aspect of the world's leading museums. However, if this readily realizable vision of data repository quality, stability and credibility is to be achieved, it will require the funders who have mandated data deposition to cover the costs of establishing and maintaining such facilities, through block grants, not through piecemeal funding to researchers. If such change is to be achieved, it must happen not only in wealthier countries but worldwide, and thus more amply provisioned funders should provide further means to help other countries improve their data-sharing capacities.

Data access is not only important post-publication, to aid reproducibility, but during peer review, so that the results of a study and their interpretations can be verified prior to publication. Providing tomographic or 3D data at the point of journal submission is, in our experience, a comparatively rare phenomenon that the publishing infrastructure is not currently well set up to facilitate. Publishers must develop a more homogeneous policy on open data [34], along with procedures to ensure data sources are acknowledged and linked electronically to the derivative publications [48]. It is also important that systems are developed to ease the submission of such data, and facilitate secure, anonymized distribution of data to reviewers. Dryad offers an integrated submission system where publishers can coordinate submission of a manuscript with submission of data, which can then be accessed securely by referees and editors. For non-integrated journals, an interim solution may be to host data at a temporary, hidden URL that can be forwarded to the reviewers via the journal. Authors may be cautious about sharing such data ahead of an article being accepted for publication, and there should be a clear policy governing the restrictions of use for reviewers.

## 8. Conclusion

Data sharing is essential in order for the benefits of 3D digital data to be fully realized by the scientific community, as well as for the maximum benefit to be gained from the public and private funding that allows these data to be collected. Not only are the benefits of 3D digital data not currently being fully realized, but failure to publish supporting data is rendering many studies based on 3D digital data at least difficult to reproduce. We have presented a series of proposals for open 3D digital data. These outline the minimal standards of verifiability that studies should meet before they are published. We also present more ambitious standards that we hope can be assumed as normal best practice (table 1). We have all been guilty of failing to meet these standards in the past because of technical and other limitations; however, technology has changed and so must we. There are costs associated with releasing data, both real and in-kind, but these are insignificant in proportion to the real costs of regenerating data, and the reputational costs to individuals, institutions, journals and editors of publishing research predicated upon inaccessible data.

## Supplementary Material

Table S1
