# Supplementary material for: Open data and digital morphology
Source: Proc Biol Sci. 2017 Apr 12;284(1852):20170194. doi: 10.1098/rspb.2017.0194 (PMC5394671; doi:10.1098/rspb.2017.0194)
Supplement: Table S1 [file rspb20170194supp1.pdf]

Table S1. Summary of main online repositories for 3D digital morphological data.

Repository	URL	Cost	Types of data files	Access rights	DOI	Journal integration	Embargo facility	Requires permission to download
Dryad	datadryad.org	$120 for up to 20 GB; $50 for each additional 10 GB	any	CC0	yes	yes	yes	no
figshare	figshare.com	Free for up to 5 GB; custom pricing for larger datasets and institutions	any	CC0 or CC BY[a]	yes	yes	yes	no
MorphoMuseuM	morphomuseum.com	free	3D models (PLY, STL, VTK) <100 MB; image stacks (ZIP) <500 MB	CC BY-NC	yes	with M3 journal	yes	no
MorphoSource	morphosource.org	free	3D models (OBJ, PLY, STL); image stacks and still images (BMP, DICOM, JPEG, TIFF)	authors' choice	yes	no	yes	authors' choice
Phenome10K	phenome10k.org	free	3D models (STL); still images of 3D models	CC BY-NC	no	no	no	no
Zenodo	zenodo.org	free	any; normally up to 50 GB per dataset	authors' choice	yes	no	yes	authors' choice

[a]May be able to choose other licenses if using institutional version.
